# Supplementary material for: Oridonin Attenuates Burkholderia cenocepacia Virulence by Suppressing Quorum-Sensing Signaling
Source: Microbiol Spectr. 2022 Jul 20;10(4):e01787-22. doi: 10.1128/spectrum.01787-22 (PMC9430380; doi:10.1128/spectrum.01787-22)
Supplement: Supplemental file 1 — Supplemental material. Download spectrum.01787-22-s0001.pdf, PDF file, 0.8 MB [file spectrum.01787-22-s0001.pdf]

Figure S1

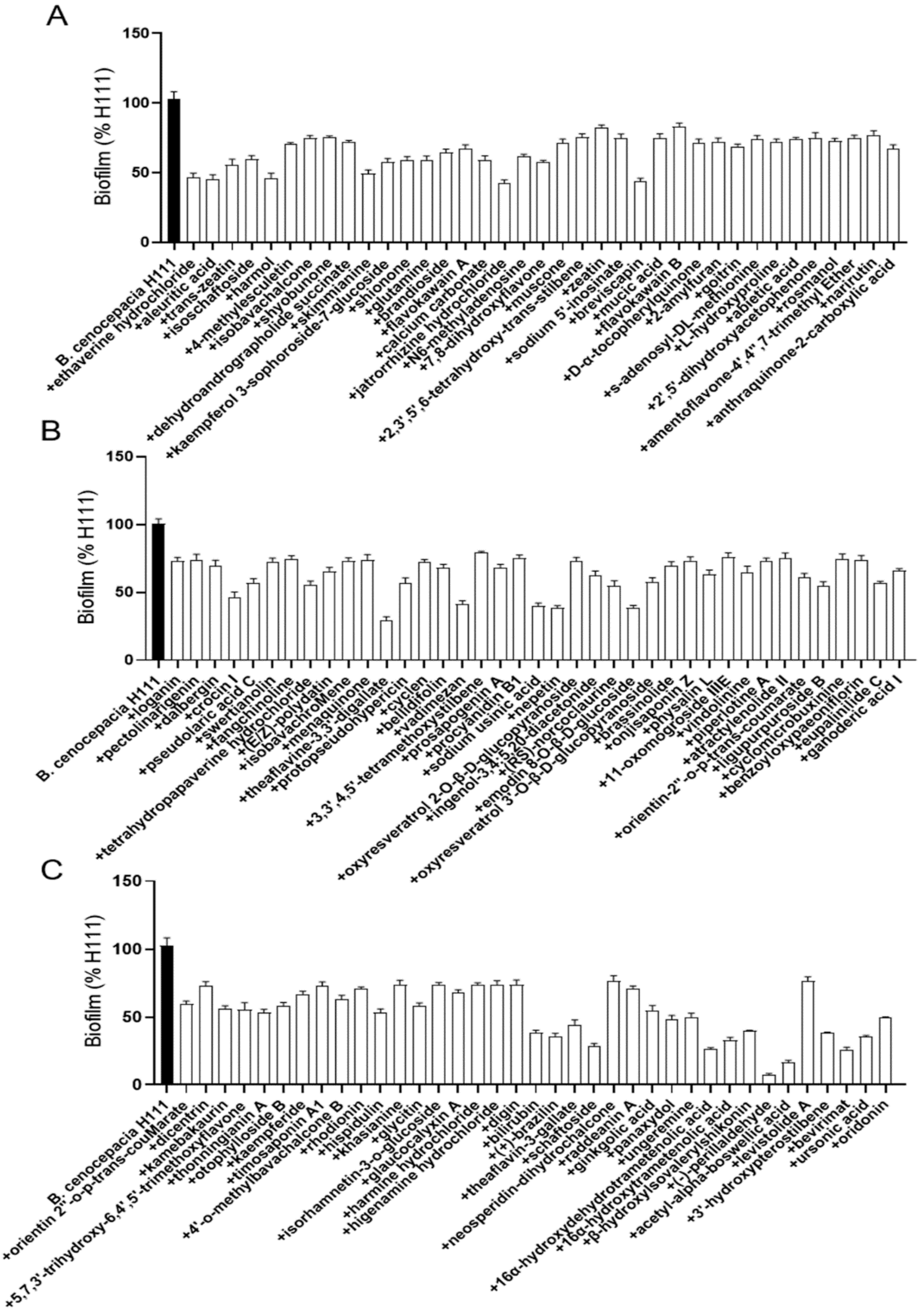

Figure S1. Biofilm formation of *B. cenocepacia* H111 in the presence of different compounds at a final concentration of 20 μM. Compounds was dissolved in DMSO, and the same volume of DMSO used as the solvent for the compounds was used as a control. The data are based on three independent experiments, and error bars represent the standard deviations.

Figure S2

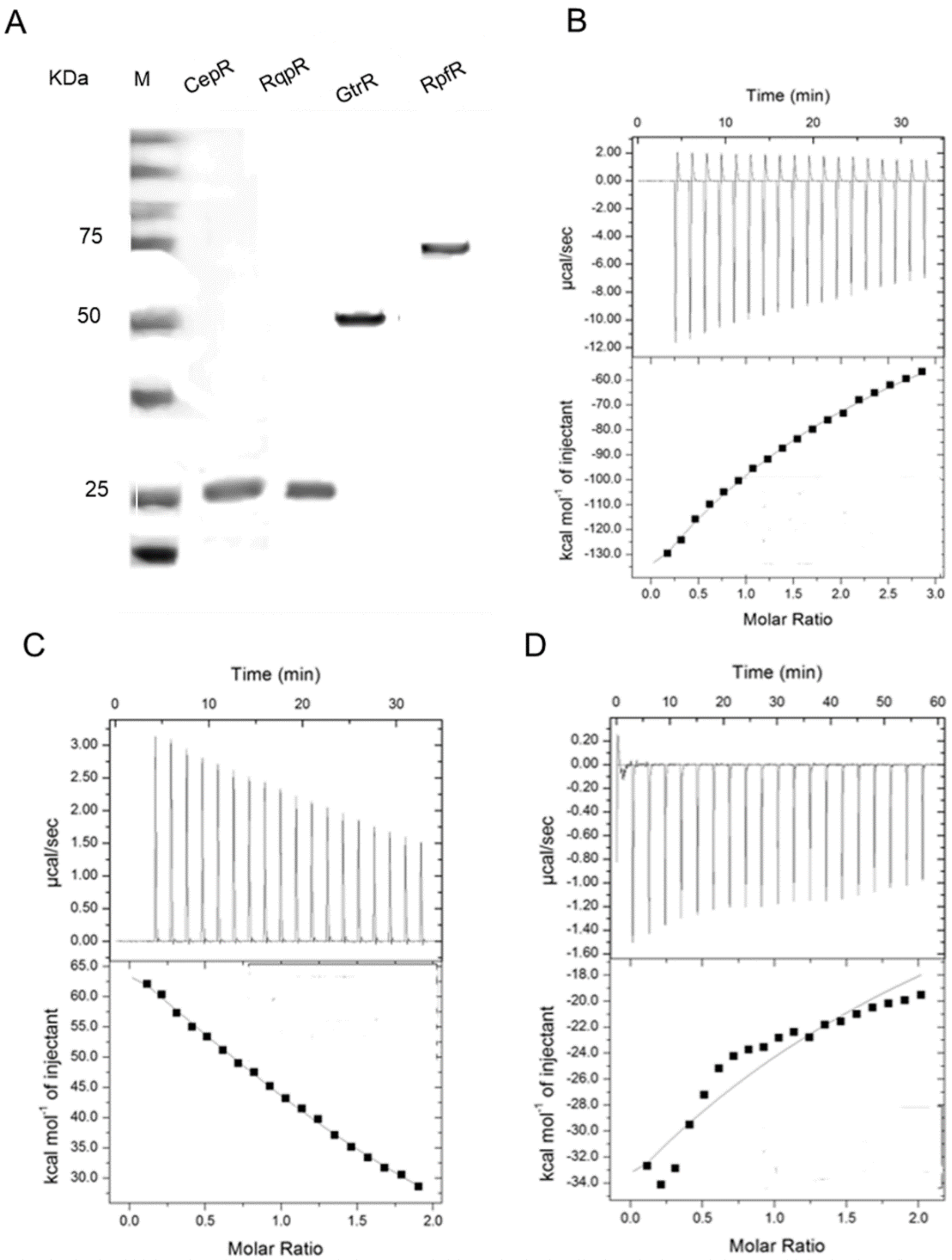

Figure S2. Analysis of the interaction of active compounds and RqpR. (A) SDS–PAGE analysis of the purified CepR, RqpR, GtrR and RpfR proteins. ITC analysis of the binding between RqpR and theaflavin-3,3'-digallate (B), thonningianin A (C), and acetyl-alpha-boswellic acid (D).

Figure S3

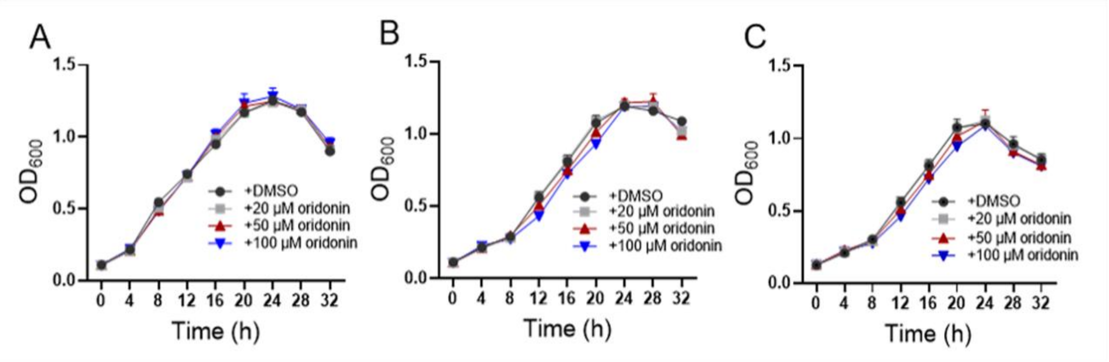

Figure S3. Effects of oridonin on the growth of *B. cenocepacia* H111 cells. The cells were grown in the presence of different concentrations of oridonin (0 to 100 μM) in LB medium (A), NYG medium (B), and MM medium (C). Oridonin was dissolved in DMSO, and the same volume of DMSO used as the solvent for the compounds was used as a control. The data are presented as the means ± standard deviations of three independent experiments.

Figure S4

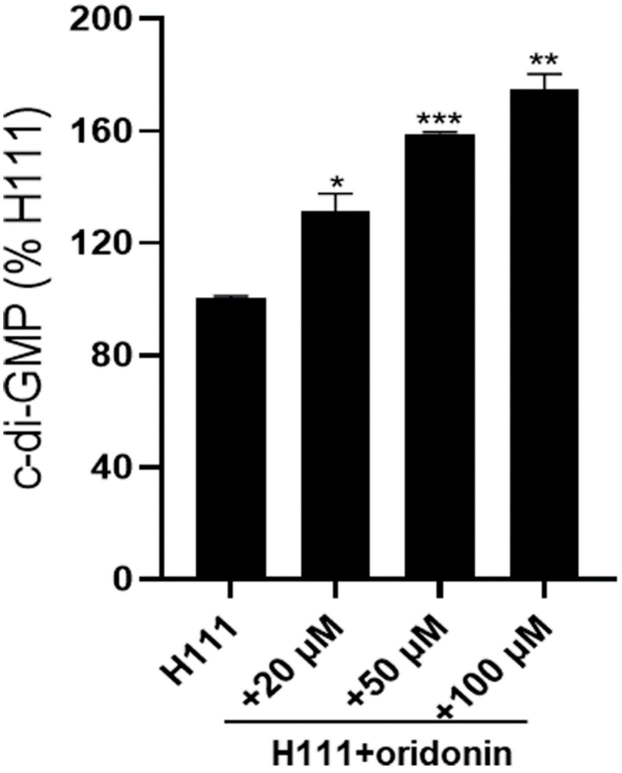

Figure S4. Effect of oridonin on the intracellular levels of c-di-GMP in *B. cenocepacia*. Quantitative analysis of c-di-GMP production in *B. cenocepacia* H111 in the presence of different concentrations of oridonin (0-100 µM). Oridonin was dissolved in DMSO, and the same volume of DMSO used as the solvent for the compounds was used as a control. For convenient comparison, the amount of c-di-GMP produced in *B. cenocepacia* H111 in the presence of DMSO without oridonin was arbitrarily defined as 100% and used to normalize the signal ratios of the samples treated with different amounts of oridonin. The data are presented as the means ± standard deviations of three independent experiments. The significance of result was determined by one-way ANOVA (*p <0.05; **p < 0.01; ***p < 0.001).

Figure S5

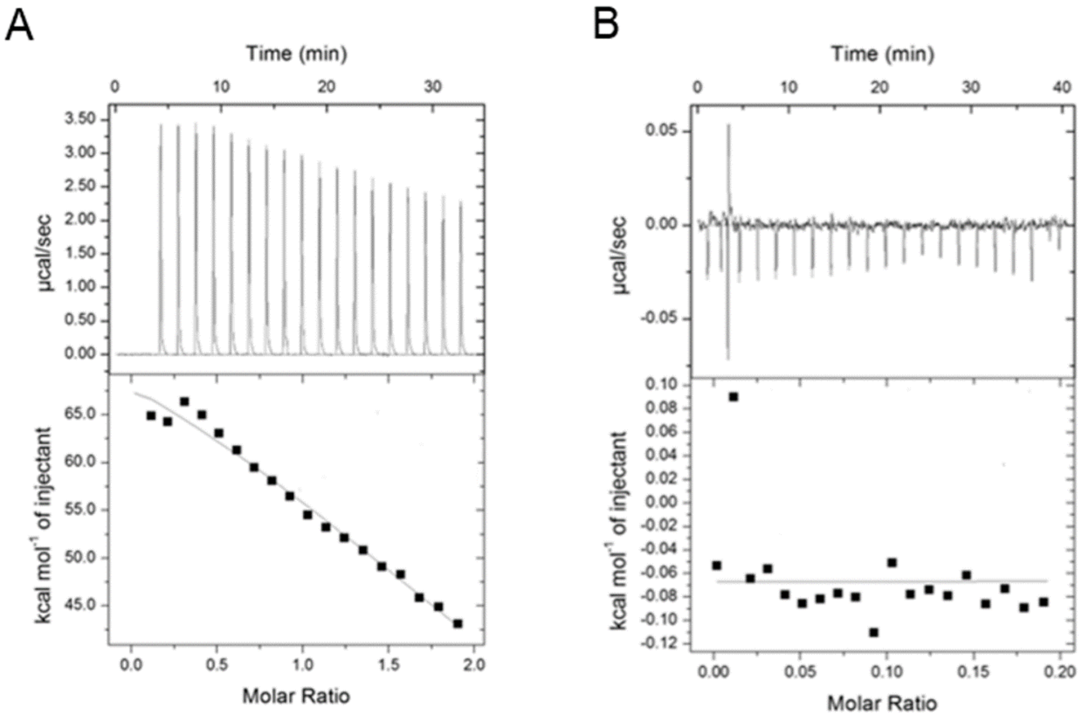

Figure S5. Analysis of the interaction of oridonin and GtrR and RpfR. (A) ITC analysis of the binding between GtrR and oridonin. (B) ITC analysis of the binding between RpfR and oridonin.

Figure S6

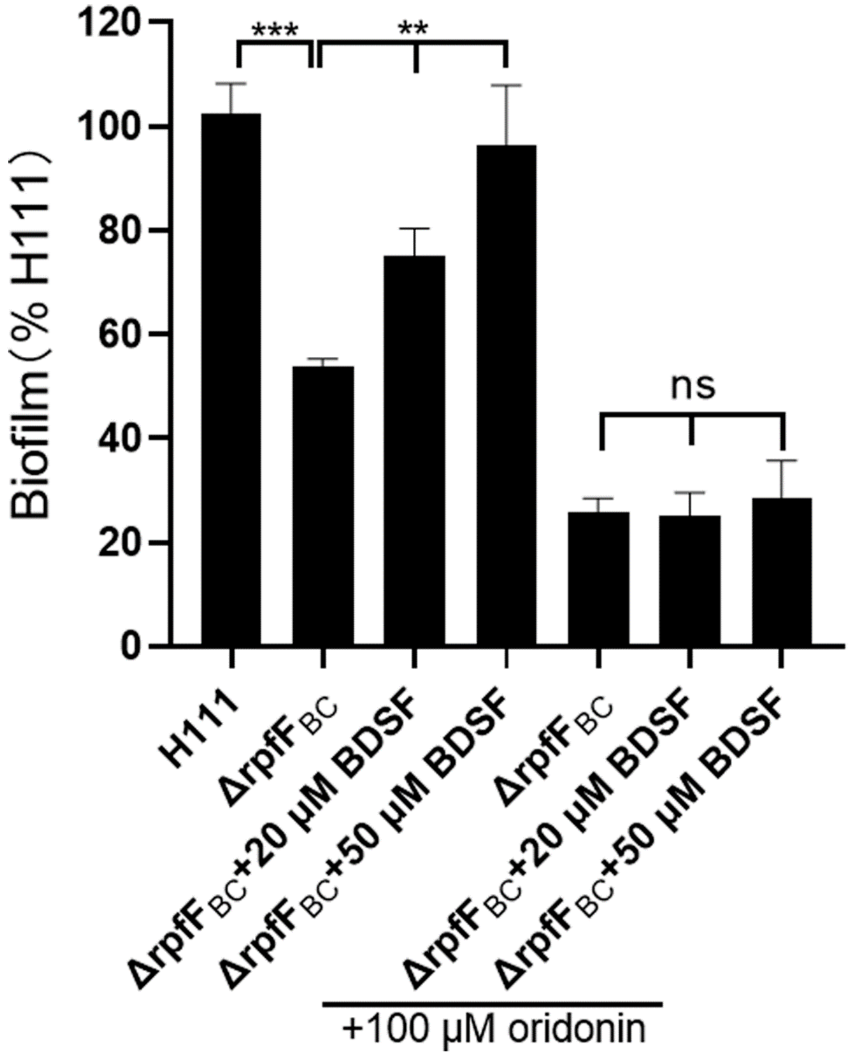

Figure S6. Effects of oridonin on the biofilm formation of *B. cenocepacia* H111 *rpfF$_{BC}$* mutant. *B. cenocepacia* H111 *rpfF$_{BC}$* mutant was treated with different concentrations of BDSF in the absence or presence of oridonin and incubated statically at 37 °C. The data are presented as the means ± standard deviations of three independent experiments. The significance of result was determined by two-way ANOVA (*p <0.05; **p < 0.01; ***p < 0.001).

Figure S7

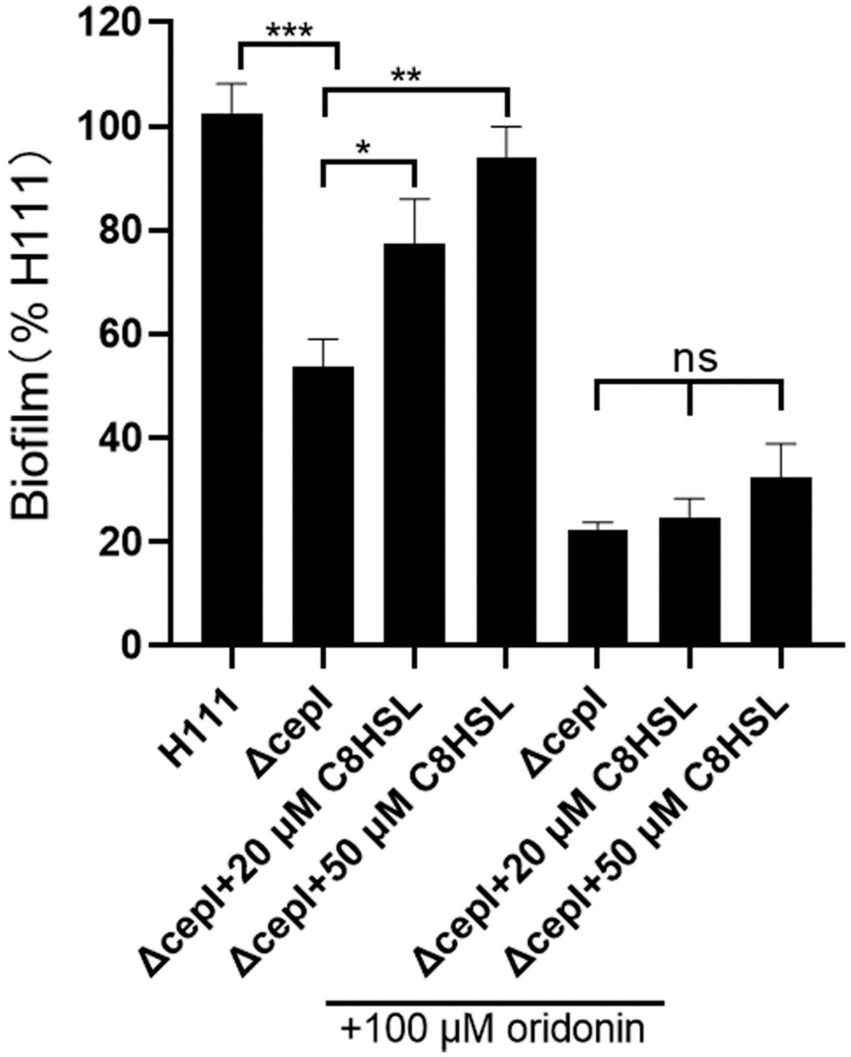

Figure S7. Effects of oridonin on the biofilm formation of *B. cenocepacia* H111 *cepI* mutant. *B. cenocepacia* H111*cepI* mutant was treated with different concentrations of C8-HSL in the absence or presence of oridonin and incubated statically at 37 °C. The data are presented as the means ± standard deviations of three independent experiments. The significance of result was determined by two-way ANOVA (*p <0.05; **p < 0.01; ***p < 0.001).

Figure S8

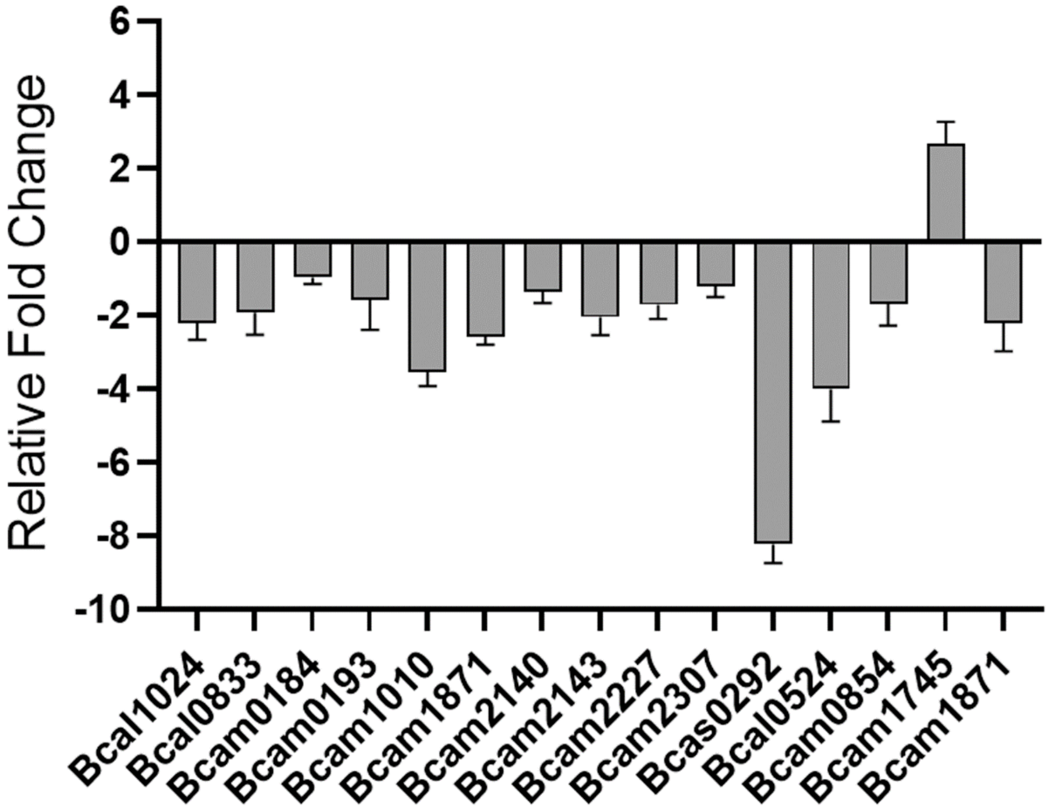

Figure S8. RT–qPCR analysis of the genes showing differential expression levels in *B. cenocepacia* H111 with addition of oridonin (100 μM). The results are based on three independent experiments. The data are presented as the means ± SD.

Figure S9

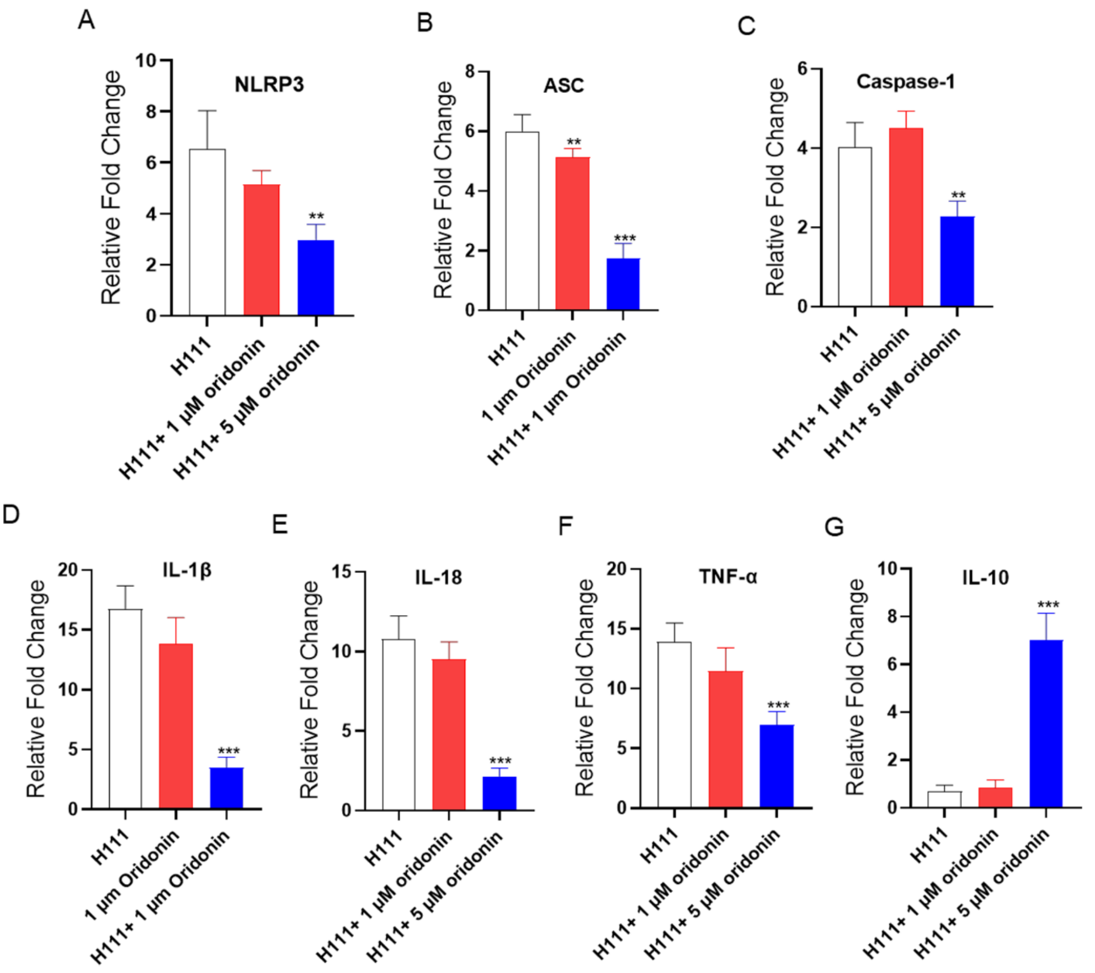

Figure S9. Effects of oridonin on the production of inflammatory factors. RAW264.7 cells were infected with *B. cenocepacia* H111 in the presence or absence of oridonin for 8 h. RT–qPCR analysis of the expression levels of NLRP3 (A), ASC (B), caspase-1 (C), IL-1β (D), IL-18 (E), TNF-α (F), and IL-10 (G). The data are presented as the means ± standard deviations of three independent experiments. The significance of result was determined by one-way ANOVA (*p <0.05; **p < 0.01; ***p < 0.001).

Figure S10

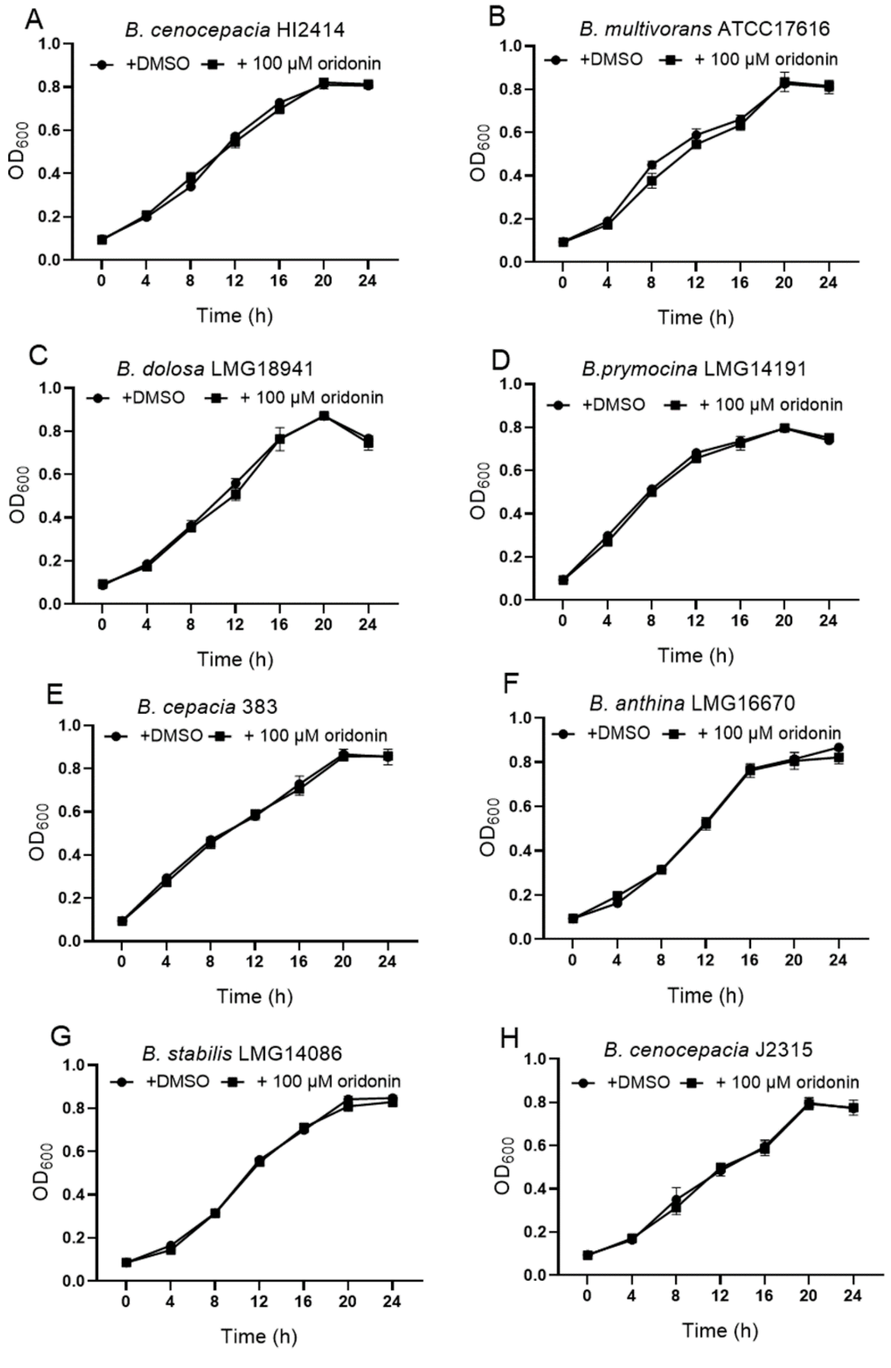

Figure S10. Effects of oridonin on the growth rate of *Burkholderia* species. Cells were grown in the presence of oridonin (100 μM) or DMSO. Oridonin was dissolved in DMSO, and the same volume of DMSO used as the solvent for the compounds was used as a control. The OD_{600} values of the samples were measured at the indicated time points. (A) *B. cenocepacia* HI2414, (B) *B. multivorans* ATCC17616, (C) *B. dolosa* LMG18941, (D) *B. prymocina* LMG14191, (E) *B. cepacia* 383, (F) *B. anthina* LMG16670, (G) *B. stabilis* LMG14086, and (H) *B. cenocepacia* J2315.

Table S1. Description of the genes influenced by oridonin

Gene	Description
Bcal1024	Transcriptional activator FlhD
Bacl0833	Acetoacetyl-Coa reductase
Bcam0184	Fucose-binding lectin II
Bcam0193	Hypothetical protein
Bcam1010	Utp–Glucose-1-Phosphate uridylyltransferase
Bcam1871	hypothetical protein
Bcam2140	Type I secretion membrane fusion protein
Bcam2143	Calcium Ion binding protein
Bcam2227	Pyochelin biosynthetic protein
Bcam2307	Metalloendopeptidase
Bcas0292	Inclusion body family protein
Bcal0524	Flagellar motor switch protein G
Bcam0854	Bifunctional exopolysaccharide biosynthesis protein (phosphomannose isomerase and GDP-Dmannose pyrophosphorylase)
Bcam1745	Putative magnesium-transporting ATPase
Bcam1871	Hypothetical protein
